# Developing Single-Molecule TPM Experiments for Direct Observation of Successful RecA-Mediated Strand Exchange Reaction

**DOI:** 10.1371/journal.pone.0021359

**Published:** 2011-07-12

**Authors:** Hsiu-Fang Fan, Michael M. Cox, Hung-Wen Li

**Affiliations:** 1 Department of Chemistry, National Taiwan University, Taipei, Taiwan; 2 Department of Life Sciences and Institute of Genome Sciences, National Yang-Ming University, Taipei, Taiwan; 3 Department of Biochemistry, University of Wisconsin-Madison, Madison, Wisconsin, United States of America; University of Minnesota, United States of America

## Abstract

RecA recombinases play a central role in homologous recombination. Once assembled on single-stranded (ss) DNA, RecA nucleoprotein filaments mediate the pairing of homologous DNA sequences and strand exchange processes. We have designed two experiments based on tethered particle motion (TPM) to investigate the fates of the invading and the outgoing strands during *E. coli* RecA-mediated pairing and strand exchange at the single-molecule level in the absence of force. TPM experiments measure the tethered bead Brownian motion indicative of the DNA tether length change resulting from RecA binding and dissociation. Experiments with beads labeled on either the invading strand or the outgoing strand showed that DNA pairing and strand exchange occurs successfully in the presence of either ATP or its non-hydrolyzable analog, ATPγS. The strand exchange rates and efficiencies are similar under both ATP and ATPγS conditions. In addition, the Brownian motion time-courses suggest that the strand exchange process progresses uni-directionally in the 5′-to-3′ fashion, using a synapse segment with a wide and continuous size distribution.

## Introduction

Faithful maintenance of genomic information is crucial for cell survival. RecA-mediated homologous recombination repairs double-stranded (ds) DNA breaks, and restarts collapsed replication forks [1], [2], [3], [4], [5]. RecA-mediated homologous recombinational repair consists of five steps: (i) polymerization of RecA onto ssDNA to form nucleoprotein filaments, (ii) searching for homologous sequences between RecA-coated ssDNA and duplex DNA, (iii) pairing with homologous dsDNA and spooling of that DNA into the RecA filaments, (iv) strand exchange between RecA-coated ssDNA and the dsDNA, with the ejection of the displaced strand, and (v) depolymerization of RecA from the heteroduplex strand exchange products [6], [7], [8]. Even with extensive studies, many mechanistic details of RecA function remain uncharacterized, mainly due to the complexity of the whole process and the strong interdependence of individual steps. In addition, the role of ATP hydrolysis in RecA reactions has been the focus of many studies. For example, even though ATP is hydrolyzed during the recombination process, RecA filament assembly itself does not require ATP hydrolysis. The end-dependent disassembly of RecA from its nucleoprotein filaments is coupled to ATP hydrolysis and represents one of the best documented roles of the hydrolytic reaction [9], [10]. ATP hydrolysis is generally required for DNA strand exchange of long DNA substrates, and it governs several mechanistic properties, such as the directionality and the capacity to bypass mismatches and other barriers [11], [12], [13]. Even though RecA-coated nucleoprotein filaments have been shown to promote limited strand exchange without ATP hydrolysis over regions of ∼1500 base pairs or less [14], [15], [16], [17] by conventional biochemical studies, how the strand exchange reaction proceeds without ATP hydrolysis is still not clear.

Recent single-molecule work by Fulconis et al [18] using magnetic tweezers offers insights on the RecA-mediated strand exchange process in the presence of torsional force. These workers showed that a three-strand intermediate occurs during the joint-molecule formation, and the displaced, exchanged ssDNA is transiently wrapped around the heteroduplex DNA. However, no recombination product was observed in the absence of negative supercoiling of duplex DNA [19]. Since DNA recombination does not always occur in specific supercoiling states, we set out to develop new single-molecule experiments to investigate the RecA-mediated pairing and strand exchange process in the absence of any external force and torsional stress. Using two different but complementary tethered particle motion (TPM) based experiments, we directly monitored the fates of both invading and outgoing strands during successful RecA-mediated DNA strand exchange reactions in the absence of external forces. For relatively short DNA substrates (a few hundred base pairs), we demonstrated that strand exchange reactions proceed efficiently with a similar rate and efficiency in the presence and absence of ATP hydrolysis. In addition, experimental time-courses suggest that the synapse progresses uni-directionally from the 5′ end to the 3′ end, using a synapse segment with a wide and continuous size distribution.

## Materials and Methods

### Proteins and DNA substrates

**Figure 1:**
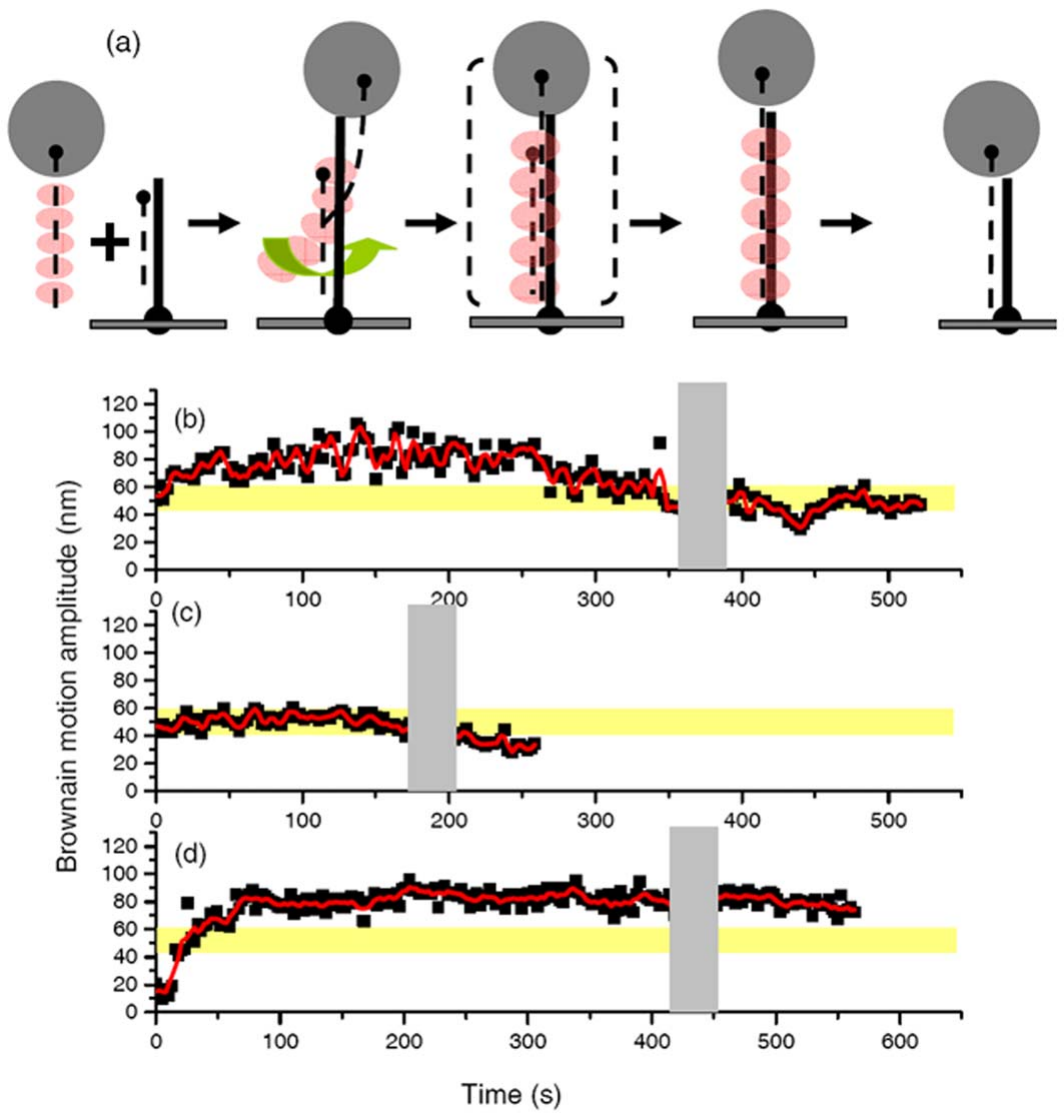
“Invading strand” experiment indicates the pairing and strand exchange process mediated by RecA recombinases. (a). Experimental design. The surface-bound DNA is the hybrid 427/352 nt DNA. The longer DNA strand has 40 and 35 nucleotides of single stranded DNA at the 5′ and 3′ ends, respectively. The invading ssDNA is fully complementary to the long ssDNA bound to the surface, and is labeled with a streptavidin bead at its 5′ end. The arrowhead indicates the 5′ end of DNA strand. (b). Time-course for successful strand exchange. The gray shaded area around 350–380 s indicates the buffer wash. The Brownian motion amplitude of the final product of duplex 427 bp DNA is shown by the yellow shaded bar. (c). Successful RecA-mediated strand exchange reaction using the shorter 229/149nt hybrid DNA (with 40 nts of ssDNA on each end) on the surface with the invading 229 nt ssDNA. The final product is fully 229 bp dsDNA, with a smaller Brownian motion. (d). RecA-mediated strand exchange reaction using 427/352 nt DNA substrate with ATPγS as cofactor. The Brownian motion remains at a higher BM value even after extensive buffer wash.

*E. coli* RecA protein was purchased from New England Biolabs (NEB) without further purification. To generate different lengths of DNA substrates with single-stranded (ss) DNA gaps at the ends, we prepared each DNA strand separately, and then annealed both strands together. Hybrid DNA substrates were designed to enhance the invasion frequency of RecA/ssDNA nucleoprotein filaments as well as to relax torsional strength generated during strand exchange process in single-molecule experiments. Each individual single-stranded DNA with hundreds of nucleotides was first prepared using a typical PCR reaction employing a 5′-phosphorylated primer and a 5′ non-phosphorylated primer (labeled with digoxigenin, biotin, or hydroxyl), followed by a lambda exonuclease (NEB) digestion at 37°C for 3 hours. Since lambda exonuclease preferentially removes mononucleotides from a 5′-phosphorylated strand of duplex DNA, this digestion results in a single-stranded DNA in which the 5′ end is not phosphorylated. PCR reactions were carried out using Phusion polymerase (NEB), pBR322 plasmid, or its derivative, 3χH3χF [20], as template. Complete sequences of primers are listed in the supplemental material (Table S1 and S2). Around 10 µg duplex DNA was digested with 50 units Lambda Exonuclease (NEB) in 100 µL reaction volume at 37°C for 4 hours. Digested ssDNA products were further verified by gel electrophoresis and gel purified before annealing to form the hybrid DNA substrates used in the experiments. Annealed gapped hybrid DNA substrates were verified by gel electrophoresis and gel purified. The homologous hybrid DNA substrates used here include (1) a 427/352 nt DNA, with a 40 nt and 35 nt gaps at the 5′ and 3′ ends, respectively, of the longer DNA strand, and (2) a 229/149 nt DNA, with 40 nt gaps at both ends (**Fig. 1a**, circles represents the 5′-end of the DNA strand). A 437 nt nonhomologous ssDNA with the 5′-end biotin-labeled is prepared with the same procedure.

### Single-molecule TPM measurement and data analysis

Streptavidin-coated beads and the coverglass reaction chambers were prepared as previously described [21]. The Brownian motion of tethered beads was observed by an inverted optical microscope (IX-71, Olympus) through Differential Interference Contrast (DIC) imaging. The images of invading strand experiments were acquired by a CCD camera (Cascade 512B, Roper Scientific) with a data acquisition rate of 16 Hz; those of outgoing strand experiments were acquired by a Newvicon camera (DAGE-MTI) at 30 Hz. Both types of experiments used custom software written in Labview. Both cameras return the same bead Brownian motion amplitude, even with a difference in their acquisition frequency. The determination of the bead centroid positions and the following data analysis were the same as described previously [22]. Brownian motion (BM) amplitude is represented by the standard deviation of the bead centroid positions of 40 frames. In order to describe DNA tether length using the bead BM amplitude, sufficient time must be given to allow the bead/DNA complex to fully explore possible configurations. Different frame durations were tested, and 40 frame durations were chosen to ensure a faithful description for DNA lengths shorter than 2000 base pairs [23], [24]. The time resolution for invading strand experiments is 2.6 s (40 frames×66 ms), and for outgoing strand experiments is 1.3 s (40 frames×33 ms).

For invading strand experiments, the digoxigenin-labeled hybrid (229/149 nt, 427/352 nt) or fully duplex (427 bp) DNA is immobilized specifically on the an anti-digoxigenin coated coverslip surface. A chamber volume (30 µl) of 20 µg/mL anti-digoxigenin (diluted with RecA reaction buffer containing 25 mM Tris-HCl, 3 mM potassium glutamate, 10 mM magnesium acetate, 5 mM DTT, 5% glycerol without BSA) was incubated on a coverslip surface for 30 min at room temperature. Three chamber volumes of RecA buffer supplemented with 1 mg/mL BSA was flowed into the chamber to remove excess, unbound anti-digoxigenin. Less than 1 nM specific DNA substrate molecules were flowed in to anchor to coverslip surface. RecA-coated ssDNA (229 nt or 427 nt) nucleoprotein filaments were prepared by incubating RecA (2 µM or 290 nM) and biotin-labeled homologous ssDNA substrates (1 nM–500 pM in total molecules), ATP (2 mM or 500 µM) and streptavidin beads (300 pM) in reaction buffer containing 5 mM dithiothreitol (DTT), ATP regeneration system (1 mM phosphoenolpyurvate and 4 units/ml pyurvate kinase) in a 37°C water bath for a half hour. Reaction variables are specified in each experiment, such as ATP concentration (2 mM or 490 µM), RecA concentration (2 µM or 290 nM), pH (pH = 7.5 or pH = 6.5), and cofactor (ATP, ATPγS or ADP) used. For reactions carried at pH 7.5, the reaction buffer was prepared based on Tris (tris(hydroxymethyl)aminomethane) hydrochloride, and for pH 6.5 reactions, ACES (N-(2-acetamino)-2-aminoethanesulfonic acid) acetate was used. Invading strand reactions were initiated by flowing in the RecA/ssDNA/bead complex attached to streptavidin-coated beads using a micropipette. The flowing time was around 20 second, and the images recorded in this period were too blurred to analyze. These complexes carried out homologous pairing with the surface-bound DNA. Tethers appeared as RecA/ssDNA/bead complex paired with hybrid DNA immobilized on the coverslip surface, and occurred at different times after reaction initiation. The reactions were quenched by flowing 100 µL reaction buffer but without the required cofactors (ATP or ATP analog) and RecA. Upon buffer wash to remove excess RecA and nucleotide cofactors, only those invading ssDNA/bead complexes that have already finished the pairing search and have completed the strand exchange step will stay in the reaction chamber as stable tethers. The completion of successful strand exchange is confirmed by the BM amplitude of expected product length of fully duplex DNA (427 bp), obtained from the control experiments (**Fig. S1**).

For the outgoing strand experiments, the hybrid DNA is digoxigenin and biotin labeled, and is immobilized at the anti-digoxigenin coated coverslip surface. Later, 300 pM streptavidin-coated beads were flowed into the chamber to attach to the distal (free) end of the digoxigenin-biotin labeled DNA. Complementary ssDNA molecules with 5′-OH ends were pre-incubated with 2 µM RecA and 2 mM ATP (or ATPγS) in a reaction buffer containing 5 mM DTT at 37°C for a half hour before flowing the complexes into the reaction chamber. The completion of RecA-mediated strand exchange reaction is signaled by the disappearance of tethered beads, indicating that the invading ssDNA has paired with and displaced the outgoing strand labeled with the streptavidin-coated beads.

For time-courses of BM amplitude in both invading and outgoing strand experiments, we identified a plateau region with a high BM amplitude. The plateau was defined by a peak at higher BM values in the histogram of individual time trajectories. Gaussian fitting of the high BM value peak produced a mean and a standard deviation. The rising dwell time (τ) was defined as the time between the initiation of an individual tether and the time point where the BM increased up to within one standard deviation less from the mean BM of the plateau. A similar analysis was carried out to identify the bead disappearance dwell time (τ_S_) in the outgoing strand experiments.

For identification of transient tethers in the invading strand experiments, only tethers persisted longer than 200 ms were analyzed. Based on Stoke-Einstein equation, we estimated that a pure diffusion process would allow 200 nm polystyrene beads to stay within the focus plane for less than 100 ms. Therefore, the 200 ms cutoff time is sufficient to identify specific interacting tethers.

## Results

We have developed two complementary, force-free single-molecule experiments based on the tethered particle motion (TPM) method to directly monitor RecA-mediated DNA strand exchange reactions. TPM experiments measure the amplitude of bead Brownian motion (BM) indicative of the tethered DNA length in the absence of force [25]. A longer DNA tether leads to a larger amplitude of bead Brownian motion, with a nearly linear correlation [23], [24]. Therefore, TPM experiments offer direct observation of the DNA tether length change during enzymatic processes at the single-molecule level. The “invading strand” TPM experiment monitors the bead-labeled RecA/ssDNA filaments interacting with homologous duplex DNA molecules linked to a cover slip, offering information on the initiation of homologous pairing and strand exchange. In the “outgoing strand” TPM experiment, the displaced strand of the target duplex DNA is tethered to the bead, allowing a direct monitoring of the completion of the strand exchange process.

### Invading strand experiment to monitor RecA-mediated strand exchange process

In the RecA-mediated three-strand exchange process, RecA molecules first bind to ssDNA to form a nucleoprotein filament, which then pairs and promotes the strand exchange reaction with a homologous duplex DNA. To monitor this process in real-time at the single-molecule level, we designed an “invading strand” experiment illustrated in **Fig. 1a**. In this experiment, duplex DNA molecules with short ssDNA extensions, 35–40 nts on both ends were immobilized onto the glass surface through a specific digoxigenin linkage on the 5′ end. The ssDNA extensions in the surface-bound duplex DNA were specifically designed to increase the invasion frequency of the RecA nucleoprotein filaments as well as to relax torsional strength generated during invasion and strand exchange processes, since experiments using DNA without the ssDNA extensions exhibited very low strand exchange efficiency (**Table 1**). Single-stranded DNA, whose sequence is complementary to the longer strand of the surface-immobilized DNA, was labeled with a 200 nm-sized bead at its 5′ end using a biotin-streptavidin linkage. The ssDNA/bead complex was pre-incubated with excess RecA in the presence of a particular nucleotide cofactor before reaction initiation. As RecA nucleoprotein filaments search and pair with the homologous duplex DNA molecules, DNA tethers appear at different initial bead BM amplitudes indicative of the initial attachment position of the nucleoprotein filament and the surface DNA (data not shown). As the RecA-mediated recombination process proceeds, the time-course of the bead Brownian motion, which reflects the change in DNA tether length, provides information on reaction progress, such as the pairing step, strand exchange step and its direction. The final recombination product is a fully duplex DNA molecule bound to the surface with one strand attached to a polystyrene bead. Knowing the length of the product duplex DNA (fully 427 bp) and its corresponding BM value, 50.5±6.5 nm (N = 175) (**Fig. S1**), the completion of the successful RecA-mediated strand exchange process can be unambiguously verified. Experiments using fully duplex DNAs with 5′-digoxigenin and 5′-biotin/streptavidin-linked bead in the presence of RecA and the non-hydrolyzable ATP analog, ATPγS, were separately carried out to identify the maximum and the final Brownian motion values possible for this recombination process (**Fig. S1**).

**Table 1:** Outgoing strand efficiency of 427/352 nt hybrid with 427 nt complementary single-stranded DNA after 15 minutes.

[RecA]	ssDNA	Nucleotide (2 mM)	Efficiency^a^
2 µM	Homologous	ATP	0.19±0.03 (N = 5)^b^
		ATPγS	0.18±0.01 (N = 5)
	Heterologous	ATP	0.08±0.04 (N = 3)
		ATPγS	0.06±0.02 (N = 3)
2 µM	Homologous	None	0.03±0.03 (N = 3)
None	Homologous	ATP	0.05±0.02 (N = 3)
None	None	None	0.02±0.01 (N = 3)

a Efficiency is defined as the ratio of disappearance events after flowing RecA with completed reaction reagent to the total observed events occurs at the time zero.

b N value refers to the number of independent experiments, with each experiment includes ∼>100 DNA tethers at time zero.

As a control to verify that our TPM experimental design faithfully monitors the RecA assembly and recombination process, we monitored the DNA length change upon RecA assembly using hydrodynamic measurements [22]. Different hydrodynamic flow rates apply different stretching forces to a DNA-bead complex. Force-extension curves of a bare DNA and a fully RecA-coated nucleoprotein filament in the presence of ATPγS can then be measured (**Fig. S2**). Consistent with the earlier report [26], upon RecA assembly, the contour length increased ∼1.7 fold as well as the dramatic increase in filament stiffness as illustrated by the persistence length changing from 44.6±12.2 nm to 739.7±150.0 nm, when the force-extension curve was fitted to the worm-like chain model (**Fig. S2**). This indicated that RecA indeed assembled onto dsDNA and formed a nearly, fully RecA-coated nucleoprotein filament. The bead Brownian motion of the same bare DNA and the RecA-DNA nucleoprotein filaments were also measured in the absence of hydrodynamic flow, returning the BM values of 83.7±7.5 nm and 180.0±35.3 nm for DNAs with lengths of 836 bp. Based on the empirical BM value-DNA length calibration curve, the length extension effect of RecA filament on these dsDNAs should produce a BM of 120.1±10.8 nm. Since the observed increase of Brownian motion amplitude (180.0±35.3 nm, N = 51) is higher than that predicted (120.1±10.8 nm, N = 85), RecA molecules not only lengthen the DNA, but also stiffen the filament in the TPM measurement.

There were two categories of synaptic events occurring once the reaction mixture was flowed into duplex DNA modified reaction chamber. One category is the transient synaptic events, in which tethers appeared briefly (a few seconds) and then detached from surface, likely due to the failure at the homology pairing. The other category is the stable synaptic events, in which tethers persistently stayed after extensive buffer wash. These tethers are successful at homology pairing and stepping into strand exchange process, and represent about ∼15–20% of the population, depending on reaction conditions (see Table 2). Out of these stable synaptic events, most (∼65±14%) led to a successful strand exchange product, as verified by the final BM value. While the other stable synaptic events did not lead to final successful products, they were stable and persistent for long time, significantly different from the transient synaptic events. These events are likely the reaction intermediates.

**Table 2:** Invading strand efficiency of 427/352 nt hybrid with 427 nt complementary single-stranded DNA after 15 minutes.

Surface bound DNA		[RecA]	pH	[Nucleotide]		Efficiency^a^
Hybrid	Homologous	2 µM	7.5	ATP	2 mM	0.17±0.06 (N = 9)(0.21±0.04)^b^ (N = 7)^c^
					500 µM	0.09±0.03 (N = 6)
				ATPγS	2 mM	0.12±0.04 (N = 6)
				ADP	2 mM	0.03±0.01 (N = 3)
		290 nM	7.5	ATP	2 mM	0.11±0.05 (N = 6)(0.13±0.02)^b^ (N = 6)
			6.5			0.17±0.02 (N = 6)(0.19±0.01)^b^ (N = 6)
		None	7.5	ATP	2 mM	0.01±0.003(N = 3)
	Heterologous	2 µM	7.5	ATP	2 mM	0.01±0.002 (N = 3)
				ATPγS		0.05±0.03 (N = 3)
dsDNA	Homologous	290 nM	6.5	ATP	2 mM	0.05±0.02 (N = 6)(0.07±0.02)^b^ (N = 6)

a Efficiency is defined as the ratio of the number of stable events to number of total observed events including transient unsuccessful process.

b Values inside bracket are from 229/149 nt hybrid substrates after 10 minutes.

c N value refers to the number of independent experiments, with each experiment includes total of ∼>200 observed events (stable and transient).

Analyzing the BM time-courses of these successful reactions indicated two types of BM patterns: In one, there is a significant rise in BM, an apparent plateau, followed by BM decrease to final product value (type I in **Fig. S3a**, ∼60%). The other type of BM time-course does not exhibit a significant rise in BM, but the BM value fluctuates around a rather constant and small BM value typical of the expected product (type II in **Fig. S3b**, ∼40%). Since both types of BM time-courses are shown to proceed to final product values (427 bp fully duplex), verified by the BM value (yellow bar) after the buffer wash (gray area), both type I and type II tethers are successful in the strand exchange reaction. A typical successful reaction BM time-course (type I) is shown in **Fig. 1b**. In this case, a surface bound 427/352 nt hybrid DNA molecule with 40 nt and 35 nt ssDNA overhangs at its 5′ and 3′ ends, respectively, was paired with a 427 nt long complementary invading ssDNA/bead complex. The zero time point corresponds to the appearance of the individual tethered bead indicating the initiation of pairing and formation of a stable synapse. For this tether, the bead Brownian motion amplitude increased from ∼53 nm to a rather constant value (a plateau) of ∼82.5±12.3 nm in the first 47 seconds. The plateau in the BM time-course was identified by a high BM value peak in the histogram (see Materials and Methods).

In the presence of ATP regeneration system and excess RecA, RecA molecules can stay on the product DNA strand after a strand exchange reaction involving circular DNA molecules [27], [28], [29]. In these experiments with short linear DNAs, a decrease in BM is noted after 250 seconds of reaction, which we attribute to dissociation of RecA filaments. To verify whether the recombination is successful, an excess buffer wash, shown by the gray bar (∼360–390 s in **Fig. 1b**), is used to remove free RecA molecules as well as to remove any remaining bound RecA molecules. After the extensive buffer wash, the RecA disassembly from the DNA was completed, leading to a decrease in bead Brownian motion to a final tethering value consistent with the expected 427 bp fully duplex DNA product (50.3±6.5 nm, shown in the yellow shaded area). Since tethers appear independently in the invading experiment, the buffer wash occurred at different time points for each tether undergoing RecA-mediated strand exchange.

To further confirm that the observed pattern in **Fig. 1b** reflects the RecA-mediated pairing and strand exchange process, we constructed similar DNA substrates but with shorter DNA lengths. Shorter DNA substrates are expected to carry out the RecA-mediated process faster with a shorter DNA product, but should follow a similar BM pattern. The reaction time-course for a RecA-coated 229 nt ssDNA/bead complex and 229/149 nt surface-bound hybrid DNA is shown in **Fig. 1c** with the similar BM pattern observed earlier in longer DNA substrates (62.5% out of total 60 tethers observed). A smaller increase in Brownian motion, occurring within 30 seconds and with a lower plateau value of 52.3±6.5 nm, is evident. The tethering value of the final DNA product of this tether was 31.0±3.2 nm, consistent with the expected, fully duplex 229 bp DNA product (29.5±4.0 nm, **Fig. S1**), confirming it as a product of successful RecA-mediated strand-exchange. Time-courses of Brownian motion amplitude change in **Fig. 1b** and **1c** share several similarities: a steady, initial increase of Brownian motion, followed by a period with an apparent plateau, and finally a gradual decrease of Brownian motion to the expected final product value. On the other hand, the maximum Brownian motion amplitude, the time required to reach the plateau, and the dwell time at the maximum BM amplitude were typically longer and larger for the longer DNA substrates (**Fig. 1b** **and** **Fig. 1c**).

**Figure 2:**
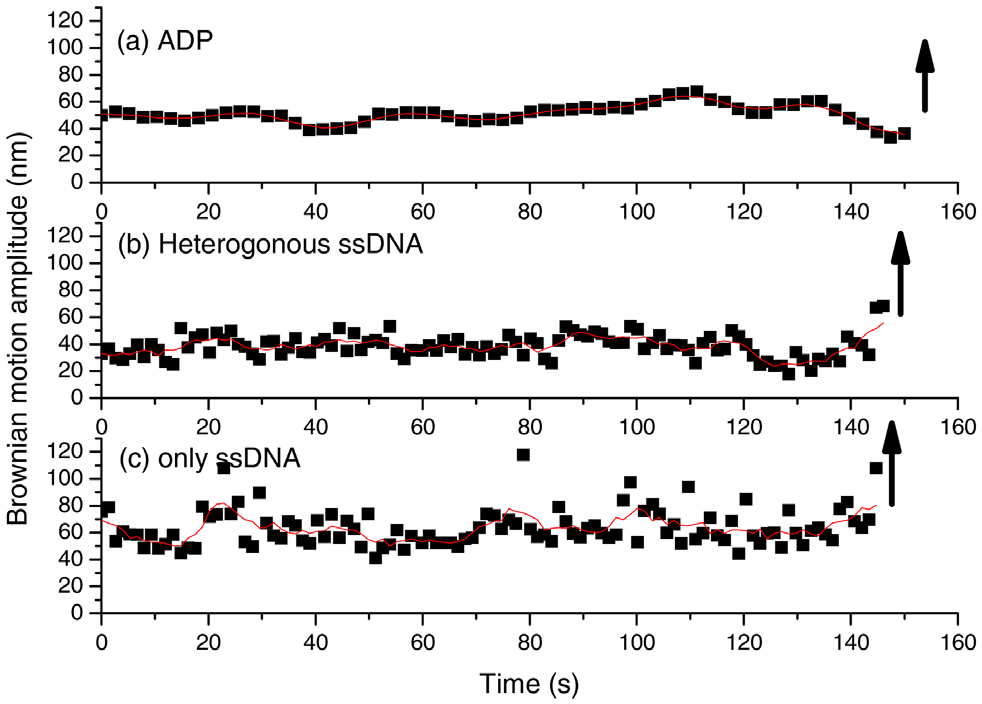
Controls for invading strand experiments with surface-bound 427/352 nt hybrid DNA. (a). Bead-labeled, homologous ssDNA substrates with ADP did not form stable tethers. (b). Bead-labeled, heterologous ssDNA. (c). Bead-labeled, homologous ssDNA substrates but without RecA and ATP. The upward arrow indicates the disappearance of the tethered bead, suggesting that the reaction did not proceed.

Control experiments, using ADP, or heterologous ssDNA in the presence of RecA and ATP or homologous ssDNA but without RecA and ATP, did not form stable tethers. Instead of forming a stable DNA tether persisting for a long period of time, most of these tethers were transient with lifetimes less than 0.2 s. Very few of these tethers (1%) (n<5) persisted for longer periods of time (>100 s), and a few of these very rare events are shown in **Fig. 2**. In all cases, the tethers later detached from the surface-bound duplex DNA, indicated by upward arrows in **Fig. 2**. This reinforces that the conclusion that the stable tethered beads observed in **Fig. 1b** and **1c** represent the successful pairing event and indeed go through the strand exchange process.

### ATP hydrolysis is not required for successful strand exchange

**Figure 3:**
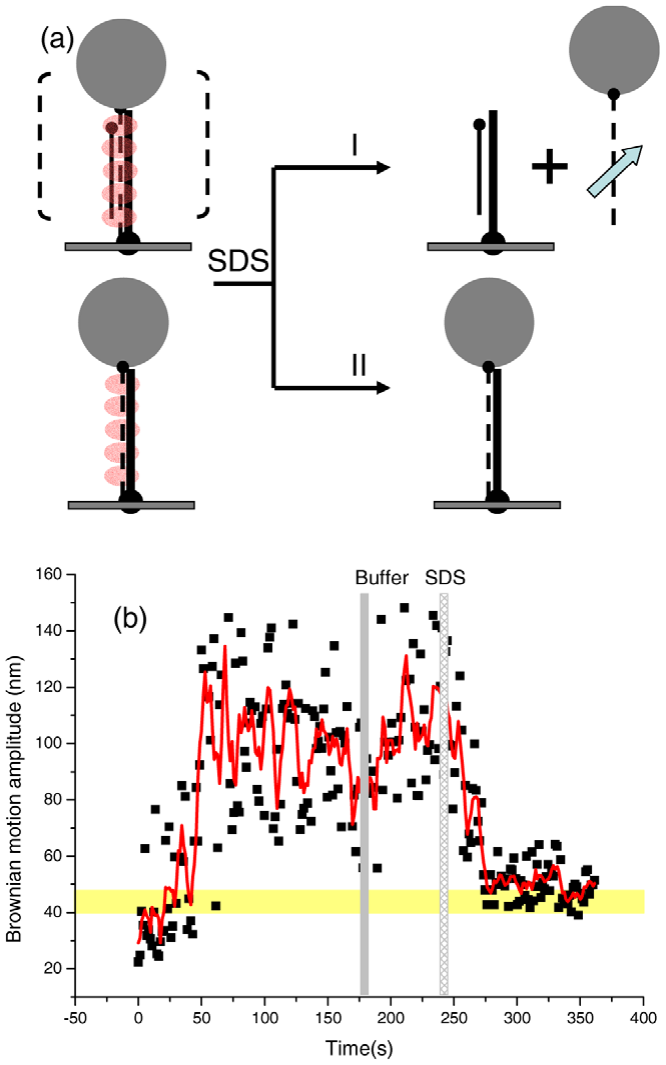
Successful completion of strand exchange reactions in the presence of ATPγS. (a). Using SDS to remove RecA from nucleoprotein filaments in order to verify the completion of strand exchange reaction in the “invading strand” experiment. (i). Tethered beads disappear after SDS addition if the RecA-mediated strand exchange reaction did not proceed. (ii). Tethered beads remain and the bead Brownian motion amplitude approaches the expected product value after SDS addition, if strand exchange reaction is successful. (b). A successful, representative time-course using 427/352 nt hybrid DNA, 427 nt homologous ssDNA and ATPγS. The tether remained after SDS addition, and the Brownian motion approaches the expected 427 bp duplex DNA value (50.3±6.5 nm).

The role of ATP hydrolysis in the RecA-mediated recombination process has been a long-lasting question in the RecA community. Here, we used single-molecule experiments to directly determine its role in different stages of the recombination process. We used a slowly-hydrolyzed ATP analog, ATPγS, to carry out an invading strand experiment. Similar to the experiments done with ATP, the reaction time-course of BM values shows an initial, steady increase and a stable plateau (**Fig. 1d**). However, even after an excess of buffer wash (∼330–370 s in **Fig. 1d**), the BM value stays at the plateau value, instead of decreasing to the final product value as in the ATP case. This plateau BM value (∼100 nm for 427/352 substrate) in the ATPγS condition is high and similar to that in the fully RecA-coated filaments (**Fig. S1e**). Earlier biochemical studies showed that RecA disassembly requires ATP hydrolysis [1], [10]. Therefore, RecA molecules are likely to stay bound to the ss/dsDNA complex in the presence of ATPγS. In other words, the assembly and homologous pairing between RecA/ssDNA and duplex DNA occur. Does the strand exchange step proceed in the absence of ATP hydrolysis? The higher BM plateau state could result from: (i) a paired homologous intermediate that does not proceed to strand exchange, or (ii) a successful strand exchange product, but with RecA bound (**Fig. 3a**). These two cases can be distinguished by removing RecA from the nucleoprotein filaments after the reaction. To test this, we applied sodium dodecyl sulfate (SDS) to remove RecA proteins from the DNA molecules after the reaction has proceeded for sufficient time to test if the strand exchange occurs. Since the DNA surface and bead/DNA anchor points are attached through anti-body/antigen linkages, which are also sensitive to SDS addition, a low SDS concentration (0.025%) was used to ensure that most of these linkages remained intact. The example time-course shown in **Fig. 3b** indicated that excess buffer wash does not reduce the plateau BM value (∼200 s, gray bar), but adding SDS (the shaded bar around 240 s) leads to a BM decrease to the expected product value (yellow row). Twenty more time-courses showed similar patterns upon SDS addition. This result directly indicates that the RecA-mediated recombination process can successfully proceed in the absence of ATP hydrolysis for the 427 bp DNA used here.

### Dissociation of the outgoing strand does not require ATP hydrolysis

**Figure 4:**
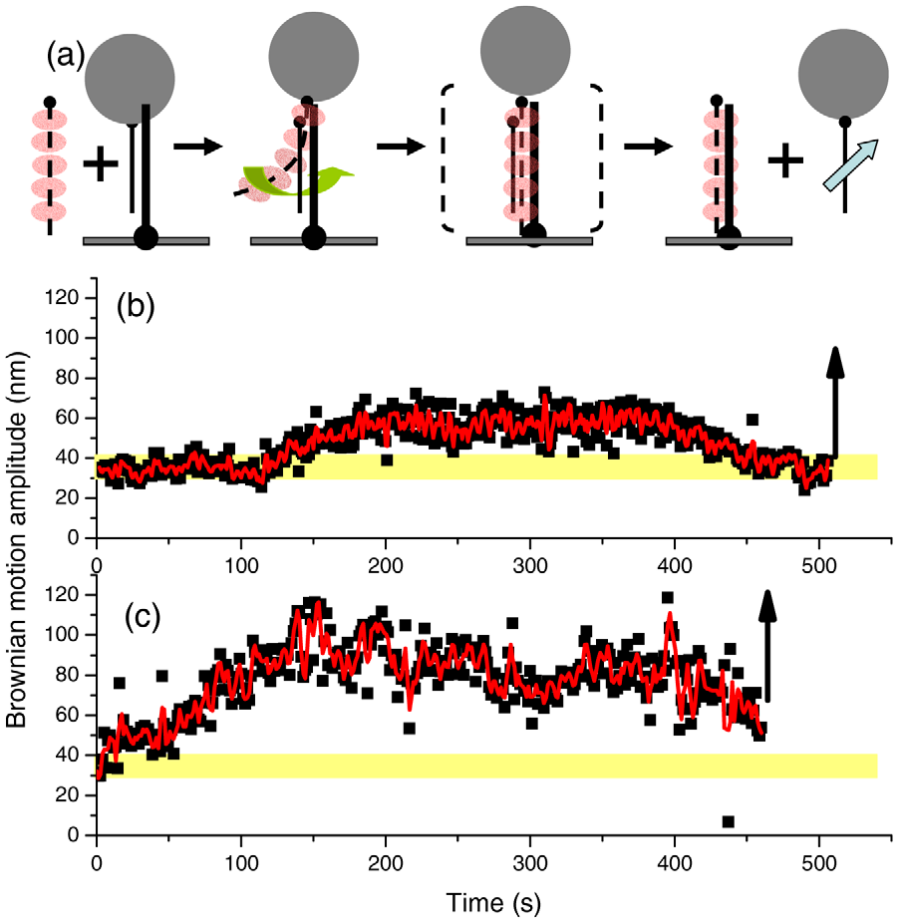
“Outgoing strand” experiments signal the completion of the strand exchange process. (a). Experimental design. Surface-bound DNA is a hybrid containing 427/352 nt DNA with a bead attached to the outgoing strand (thin line, 352 nt ssDNA). 427 nt invading ssDNA is complementary to the surface-bound hybrid DNA. The arrow indicates the 5′ end of DNA strand. (b). BM Time-course using homologous ssDNA and ATP. Beads disappeared as indicated by an upward arrow. (c). Experiments using ATPγS also show bead disappearance.

Our “invading strand” experiments directly monitor the pairing step of the RecA-mediated process, and offer details on the pairing and strand exchange process. To further confirm that strand exchange could occur in the absence of ATP hydrolysis, we designed a complementary “outgoing strand” experiment, illustrated in **Fig. 4a**. In this outgoing strand experiment, a streptavidin-labeled bead is attached to the strand that is to be displaced from the surface-bound hybrid DNA. Reactions were initiated by the introduction of pre-incubated RecA-coated complementary ssDNA. As stable synapse forms and strand exchange commences, RecA nucleoprotein filament binds to duplex DNA, and the tethering bead Brownian motion increases. Therefore, the Brownian motion amplitude of the tethering beads can provide details on the pairing and strand exchange steps. As soon as the strand exchange step is completed, one of the original duplex strands is displaced, which is indicated by bead detachment from the surface. Disappearance of the tethering beads thus provides a unique way to monitor the completion of the strand exchange reactions. Typical time-courses of the outgoing strand experiments using the 427/352 substrates are shown in **Fig. 4b** (with ATP) and **4c** (with ATPγS), with the initial BM centered around 36.3±4.7 nm (**Fig. S1c**). Both traces show a similar pattern: initial increase in bead Brownian motion amplitude, likely due to the increasing amount of RecA/ssDNA segment interacting with the surface-bound DNA, and finally, the disappearance of tethered beads, as shown by the upward arrows. Control experiments without ATP, or without RecA, or using non-complementary single-stranded DNA, or with buffer only showed no change in Brownian motion amplitude (**Fig. S4**), and a very low percentage of beads disappeared (3–5%, **Table 1**) during a 15 minute observation time. Washing extensively with buffer (without RecA and ssDNA) led to ∼2% tethered bead disappearance, probably due to the detachment of tether beads from surface nonspecifically. In contrast, a statistically much higher percentage of tethered beads disappeared in an identical 15 min observation window when the reactions were implemented with homologous RecA-ssDNA in the presence of ATP (19±3%, **Table 1**) or ATPγS (18±1%). Consistent with this observation, the time required for outgoing strand disappearance is approximately the same for both ATP and ATPγS in the outgoing strand experiments (**Fig. S5**).

### Strand exchange efficiency in invading and outgoing strand experiments

We then examined the strand exchange efficiency under a variety of conditions (**Table 1** and **2**). In the invading strand experiment, the efficiency is defined as the ratio of successful strand exchange events over the total number of observed tethers, including successful strand exchange and transient pairing. Transient tethers were defined as beads that remained in a particular position longer than 200 ms. Based on the Stoke-Einstein equation, the time for a 200 nm polystyrene bead to diffuse out of the focus plane is less than 100 ms. Therefore, the 200 ms cutoff time is sufficient to identify specific interacting RecA/ssDNA tethers. In the outgoing strand experiment, the strand exchange efficiency is defined as the ratio of disappearing tether beads to the total number of tethered beads available at time zero.

The fact that the pairing occurs in the presence of ATP and ATPγS, but not with ADP, clearly indicates that the ATP-like nucleotide state, a high affinity state, is required for both the RecA-mediated pairing and strand exchange steps. Our method thus generates results that parallel those obtained in many other studies. Strand invasion reactions using ATPγS can lead to stable tether formation with an efficiency of 12±4% of the tethers observed during a 15 minute reaction time, nearly as great as that seen in the presence of ATP (17±6%, **Table 2**). This stands in strong contrast with the 3±1% efficiency observed in the presence of ADP. This indicates that ATPγS can be an efficient cofactor, similar to ATP. The efficiency of strand exchange in the outgoing strand experiments with both ATP and ATPγS (18–19%, **Table 1**) correlated well with those in the invading strand experiments (12–17%, **Table 2**). Both experiments directly demonstrate that the RecA-mediated strand-exchange process can occur with these DNA substrates in the absence of ATP hydrolysis with similar efficiency. Significantly, strand exchange with ATPγS consistently goes to completion, with release of the displaced DNA strand in these experiments.

Since the percentage of completed strand exchange events within a 15 min reaction time window is smaller than 20%, we decided to follow the reaction for two hours to calculate the strand exchange efficiency in the outgoing strand experiment (**Fig. S6**). After two hours of reaction, the strand exchange efficiency in the presence of ATP (65±9%) or ATPγS (47±9%) is significantly higher than that for control experiments without RecA or without ssDNA (both ∼13±5%, **Fig. S6**).

Moreover, most of the disappearing tethers in the outgoing strand experiment include an apparent increased BM before disappearing (51% and 39% for ATP and ATPγS respectively, **Fig. S7**). The steady increase in BM value of the surface-bound DNA tether is likely due to the pairing/strand exchange interaction with the RecA/ssDNA nucleoprotein filament. We defined the strand exchange time, τ_s_, as the duration of the BM plateau before the bead detachment (**Fig. S5**) representing the rate of displacement of the outgoing strand. Analysis of τ_s_ returns the mean duration of 243±190 s and 211±150 s for ATPγS and ATP, respectively (**Fig. S5b**), corresponding to the strand exchange rates of 1.76 and 2.00 nt/s. Identical strand exchange rates in the presence of ATP or ATPγS are consistent with the conclusion that ATP hydrolysis is not required for RecA-mediated pairing and strand exchange for these short DNA substrates.

### The effect of sub-saturating ATP concentrations

Many studies of RecA nucleoprotein filament formation have documented the effects of nucleotide cofactors [30], [31], [32], [33]. Since the nucleotide binding site lies at the RecA-RecA subunit interface, and its binding is critical for the RecA nucleoprotein filament stability [7], [31], it is likely that stable, continuous RecA nucleoprotein filaments require sufficient ATP. To see if insufficient ATP molecules indeed alter the filament length, and in turn, alter the reaction efficiency, experiments were done at lower ATP concentrations at its K_d_ value (500 µM, **Fig. S8b**). Similar Brownian motion patterns were observed as seen in the typical experiments, except that lower BM amplitudes of the plateau (56±11 nm in the trace shown here) were observed with 490 µM ATP.

## Discussion

### RecA-mediated strand exchange proceeds in the 5′-to-3′ direction

**Figure 5:**
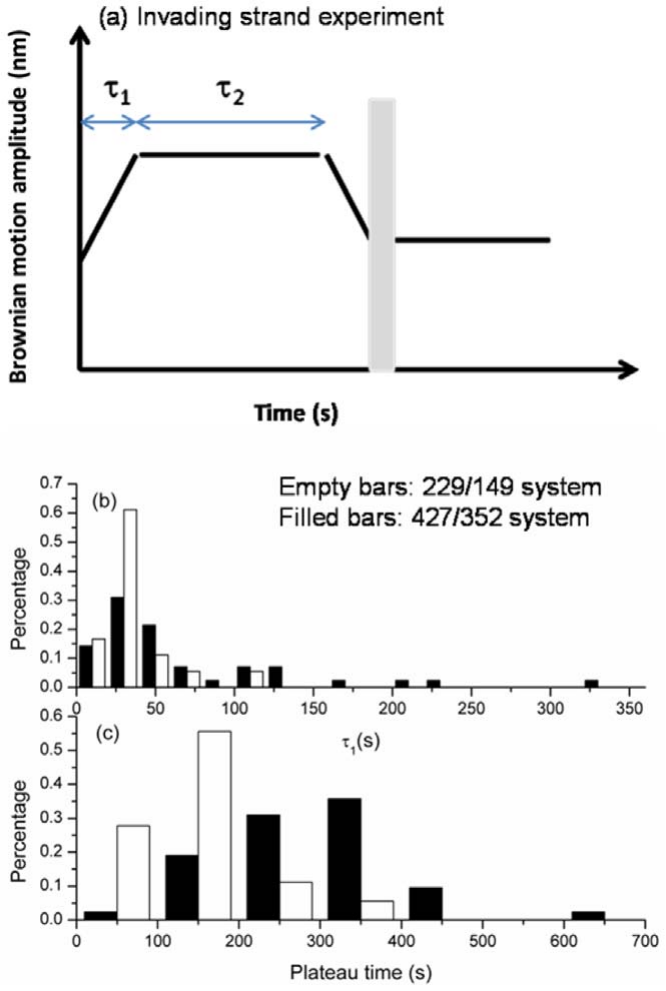
Analysis for invading strand time-courses at two DNA lengths. Empty bars represent 229/149 nt system; filled bars represent 427/352 nt system. (a). A cartoon of invading strand time-course is used to define various stages of reaction: the duration between the establishment of a tether and the beginning of the plateau is defined as τ_1_; the duration of the plateau is defined as τ_2_. Only tethers showing a BM decrease before buffer wash were used for analysis. (b). τ_1_ exhibited no apparent difference at two different DNA substrates (mean of 28.6±15.1 s and 36.7±13.3 s for 229 and 427 substrates). (c). τ_2_ histogram shows DNA length dependence for two different DNA substrates (138.6±67.7 s and 289.6±102.9 s for 229 and 427 substrates).

In this report, we illustrate the potential of a new single-molecule method for monitoring RecA-mediated DNA strand exchange. The work also reveals a few new aspects of this reaction. Time-courses of BM change in successful invading strand experiments (such as those in **Fig. 1b and 1c**) share distinct stages: a steady, initial increase of Brownian motion, followed by a period with an apparent plateau, and finally a gradual BM decrease to the product DNA value. It is thought that a bead-labeled RecA/ssDNA nucleoprotein filament first interacts with surface-bound, duplex DNA by random collision. Since this interaction can occur at any point along duplex DNA, no preferred initial BM value was identified (data not shown). Knowing the rate constant of homologous pairing is on the order of ∼10^5^ s^−1^ [34], [35], [36], it is most likely that bead-labeled RecA nucleoprotein filaments had already located the homologous sequence of the duplex DNA when the stable synaptic state formed. If RecA nucleoprotein filaments have to search for homologous sequence after the formation of tethers (either through moving upstream/downstream along DNA one-dimensionally or through random collision/dissociation three-dimensionally), BM time-courses will show an apparent fluctuation in the very beginning. However, a steady increase of bead Brownian motion right after bead tethering discounts this possibility. After forming a stable synaptic state, the pairing between the RecA nucleoprotein filament and the homologous surface-bound DNA continues. The tethered bead Brownian motion steadily increases due to increasing amount of RecA/ssDNA segment interaction with the duplex DNA and the accompanying increases in length and stiffness. As discussed earlier, the plateau duration τ_2_ shows obvious length-dependence, but the BM increase phase, τ_1_, does not (**Fig. 5**). The absence of a length-independence of τ_1_ and the uni-directional increase in BM allows us to speculate that the major event occurring within τ_1_ is the pairing between the RecA/ssDNA nucleoprotein filament and homologous duplex DNA in order to establish the minimum functional strand exchange segment. This phase includes a rapid wrapping of the duplex DNA into the filament, since the increase in BM implies an incorporation of the dsDNA into the filament. On the other hand, we speculate that the τ_2_ process reflects strand exchange itself due to its length-dependence [19]. The strand exchange rate is estimated to be 1.65 and 1.47 nt/s for 229/149 and 427/352 substrates in these invading strand experiments. These strand exchange rates are the same as determined in the outgoing strand experiments under either ATP (2.00 nt/s) or ATPγS (1.76 nt/s), as well as quite similar to estimations derived from ensemble-averaged studies under similar conditions [19], [37].

**Figure 6:**
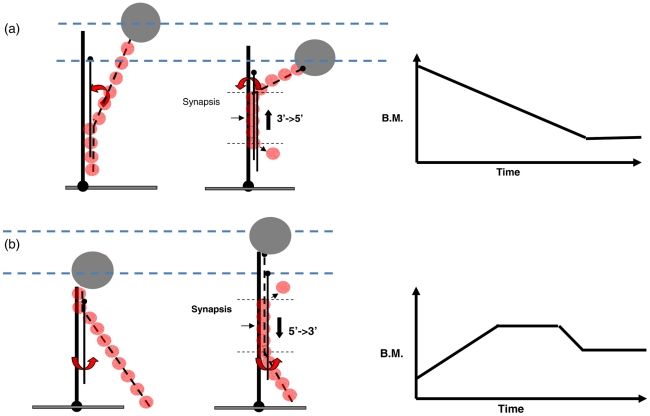
Proposed strand exchange model and the corresponded Brownian motion time-courses in the invading strand experiment. (a). Strand exchange proceeds in the direction of 3′-to-5′ and the corresponding BM time-course. (b). Strand exchange proceeds in 5′-to-3′ and the corresponding BM time-course.

When a stable tether is first established in the invading strand experiment, there are two contributions to the BM value: (1). the non-RecA-coated duplex DNA from the surface attachment point to the initial pairing point, and (2). the RecA-coated ssDNA from the pairing point up to the bead. Let us now consider the consequence of BM time-courses based on the strand exchange direction. If the strand exchange proceeded in a 3′-to-5′ direction, the initial BM amplitude will start at a rather higher value due to the major BM contribution from the RecA/ssDNA nucleoprotein filament that is already lengthened and stiffened (**Fig. 6a**). As RecA dissociates after the exchanged strand is released, the 3′-to-5′ strand exchange direction would lead to a decrease in BM. On the contrary, if the strand exchange proceeds in a 5′-to-3′ direction, the initial BM value will start low due to a major contribution from the surface-bound, unbound DNA (**Fig. 6b**). As the reaction progresses in the 5′-to-3′ direction, more RecA nucleoprotein filament interacting with duplex DNA would lead to a continuous BM increase followed by a plateau BM where a constant, fixed-size synapse segment propagates. None of the successful BM time-courses observed shows an initial BM decrease, but 60% of the time-courses observed with ATP included a steady increase at the initial stage (**Fig. S3**). The other 40% of the time-courses resulting in stable tethers showed time-courses where the BM remained at lower values (∼40–60 nm for 427/352 substrates), inconsistent with the high BM values expected from the 3′-to-5′ model. Therefore, our data is consistent with the model where the synapse progresses 5′-to-3′ in the RecA nucleoprotein filament. The directionality of the synapse progression can thus be implied from the BM pattern.

### Continuous, broad size distribution of active RecA synapse segment

**Figure 7:**
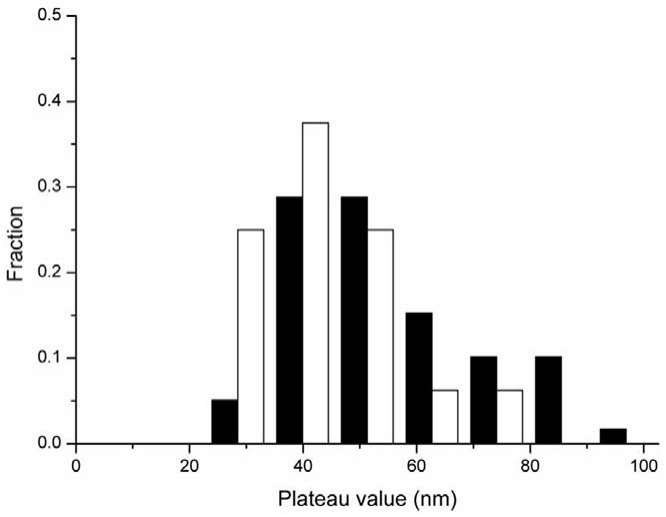
The continuous, broad size distribution of active RecA synapse segment observed in invading strand experiments. The histogram includes the plateau BM value (type I) and the mean BM value (type II) observed in 229/149 (empty bar) and 427/325 (filled bar) substrates. Both DNA substrates showed a continuous, broad BM distribution.

Stable synaptic events in our TPM invading strand experiments indicated that both the reactions with increasing-plateau-decreasing BM patterns (type I, **Figure S3a**, 60%), and the ones without apparent BM change in time-courses (type II, **Figure S3b**, 40%) succeeded in strand exchange reactions. The BM value change in the invading strand pattern reflects the size of RecA-coated segment involved during the strand exchange reaction. In the type I BM pattern, the apparent plateau reflects the size of the active RecA synapse segment involved in the strand exchange. In the type II BM pattern, the absence of an apparent change in BM value suggests involvement of a relatively short RecA segment that is not long enough to register an increase in BM in our experiments. Analyzing the combined histogram of the plateau BM value (type I) and the mean BM value (type II) shows a single continuous, broad size distribution of active RecA synapse segments (**Figure 7**). This continuous size distribution is consistent with a model that there is a range of RecA filament lengths capable of carrying out the strand exchange reactions successfully. These RecA synapse segments range from a relatively small size to long filaments that almost saturate the available DNA. Control experiments using ATPγS return with longer filaments as expected (**Fig. S9**). The involvement of shorter RecA synapse segment observed in this work is in contrast to the ∼30 RecA fixed-sized, active synapse segment suggested by *van der Heijden et. al.* [19]. However, their magnetic tweezers experiments had a limited capacity to observe reactions with apparent length changes to register as signals. Therefore, successful strand exchange reactions mediated by shorter RecA synapse segment might be missed in their experiments, but were clearly seen in our invading strand experiments. We thus propose that the length of the synapse segment of a RecA filament can vary from one strand exchange reaction to another.

Beads are attached to the invading strand in invading strand experiments, which directly monitor the pairing as well as the strand exchange process of RecA nucleoprotein filaments. In outgoing strand experiments, beads are attached to the to-be-displaced, exchanged strand. Therefore, outgoing strand experiments monitor the dynamics of the displaced, exchanged strand, and the disappearance of the bead indicates the completion of the strand exchange reaction. Surprisingly, similar to the major BM pattern observed in invading strand experiments, the type I BM pattern (51%, **Fig. S7a**) in outgoing strand experiments also includes an initial BM increase, plateau, a BM decrease, followed by the bead disappearance. Consistence between these two different experiments confirms the significance of the observed BM pattern. The BM plateau observed in the outgoing strand experiments further suggests that the displaced, exchanged strand has to be temporarily bound with heteroduplex DNA upstream of the synaptic region during RecA-mediated strand exchange process until RecA molecules eventually dissociate and the exchanged strand is displaced from the heteroduplex DNA, which is signaled by the detachment of the tethered polystyrene bead. In addition, ∼50% of successful outgoing strand tethers showed a type II BM pattern (**Fig. S7b, c, d**) with no apparent BM change before bead disappearing. This percentage is similar to what observed in the invading strand experiments (∼40%), consistent with our proposal that many reactions employ a shorter active RecA synapse segment as mentioned above.

### RecA nucleoprotein filament stability determines the strand exchange efficiency

Even in the reaction conditions favoring a successful recombination process (containing homologous ssDNA, RecA and ATP), we also observed many transient tethered beads, which appeared, persisted briefly and then detached from the surface. The presence of these transient tethers (existing for a few seconds to minutes before dissociating) suggests that an interaction between RecA nucleoprotein filaments and surface-bound DNA occurred, likely due to a search for homology. However, the dissociation happened because either no homology sequence was recognized, or full strand exchange step was not successfully initiated for these transient tethers. In a 15 minute observation time window, ∼83±6% of tethers observed (with lifetime longer than 200 ms) are transient. On the other hand, there were some tethers that persisted stably for several minutes even after buffer wash. The major portion of these stable tethers (65±14%) sustained after extensive buffer wash, exhibit BM values consistent with the expected strand-exchanged DNA product. The other tethers were apparently shorter in DNA tether length, but stable, likely reflecting reaction intermediates that are part-way through the strand exchange step. Therefore, we interpret the stable tethers that remain after extensive buffer wash as reactions undergoing complete strand exchange, so there is sufficient interaction between the invading strand and duplex DNA to form a stable complex.

Under our typical reaction condition with saturating ATP (2 mM) and excess RecA (2 µM), about 17±6% (see **Table 1**) of the tethers were stable tethers. In controlled experiments using heterologous DNA, virtually all of the tethers disappeared after extensive buffer wash. The strand exchange efficiency declined with lower RecA concentrations (11±5%) than that observed with excess RecA (17±6%, **Table 1**) while the plateau BM amplitude value and plateau duration are similar. These observations are most consistent with the possibility that limited RecA concentration still results in DNA coated to its minimum active segment, but the number of individual RecA-coated DNA filaments is reduced.

For *E. coli* RecA protein, nucleation on DNA is more efficient at lower pH values (pH<6.5) [9], [38], [39], [40]. Consistent with this observation, the RecA-mediated pairing and strand exchange processes we observed were more efficient at lower pH (see **Table 1**). The strand exchange efficiency at pH 6.5 is more efficient (17%) than that at pH = 7.5 (11%) for hybrid DNA. The difference is even more obvious for the much less efficient reactions involving fully duplex DNA molecules, where nearly no successful reactions occur at pH = 7.5, but a significant (still less than 5%) efficiency is observed at pH 6.5 (data not shown).

In the presence of ADP, the RecA-mediated strand exchange reaction did not proceed, with most tethers occurring transiently (tethering lifetime <2 s). This is consistent with the RecA nucleoprotein filament affinity states proposed by *Joseph et. al.* [31] that ATP binding, but not ADP, shifts the filaments to an affinity state that facilitates overcoming the barrier caused by repulsion between two DNA molecules and disrupting the Watson-Crick hydrogen bonding in the duplex DNA. This RecA high affinity state accounts for how the strand exchange efficiency, thus the recombination efficiency, depends on the ATP concentration shown in **Table 2**.

### Conclusion

The TPM experiments developed here provide a direct visualization of RecA-mediated pairing and strand exchange process. The two complementary TPM experiments offer a unique opportunity to monitor the fates of different DNA strands, providing a more detailed view of the reaction than existing methods. In addition, our experiments are carried out without a need to apply external force to the DNA molecules or RecA filaments. Consistent with the propensity of RecA to nucleate filament formation on DNA more rapidly at lower pH, we found that the successful RecA-mediated pairing and strand exchange reactions with these short DNA substrates are more efficient at lower pH (pH = 6.5). The reactions require a high affinity state, bound with ATP-like nucleotides. Both invading strand and outgoing strand experiments share the same BM pattern characteristics. The BM time-courses imply that the strand exchange progresses 5′-to-3′ uni-directionally. The size distribution of active RecA synapse segment is found to be continuous and broad, all capable of carrying out successful strand exchange processes. The BM plateau obtained in outgoing strand experiment indicates that the exchanged strand remains bound to heteroduplex DNA. Using the non-hydrolyzable ATP analog, ATPγS, we found that there is no difference in strand exchange rate and efficiency. Therefore, ATP hydrolysis is not required for the completion of the strand exchange reaction. Significantly, the displaced DNA strand is released even when strand exchange is carried out with ATPγS. Further single-molecule studies of the coupling between ATP hydrolysis and DNA strand exchange using circular DNA substrates will offer new insights into the molecular details of the role of ATPase in RecA-mediated strand exchange process.

## Supporting Information

Figure S1 BM histograms of various DNA substrates, expressed by the standard derivation of bead centroid position. (a). 229 bp fully duplex DNA, 29.6±4.0 nm (N = 218). (b). 427 bp fully duplex DNA, 50.5±6.5 nm (N = 175). (c). 427/352 nt hybrid DNA, 36.6±4.7 nm (N = 78). (d). Fully RecA-coated 229 bp duplex DNA under ATPγS, 67.6±14.4 nm (N = 50). (e). Fully RecA-coated 427 bp dsDNA under ATPγS, 101.3±28.8 nm (N = 56).
(DOC)

Figure S2 The force-extension curve for a 836 bp dsDNA molecule tethered with 200 nm polystyrene bead done by applying a hydrodynamic force. Solid circles represent the force-extension curve for bare 836 bp dsDNA and empty circles represent that for RecA-coated 836 bp dsDNA using ATPγS. The solid curves are fitted to a worm-like chain model with fitted parameters for persistence length of 44.6±12.2 nm and 739.7±150.0 nm; for contour length of 280.3±2.4 and 475.3±2.8 nm for bare and RecA-coated dsDNA. The force was determined from the mean-squared displacement (MSD) of beads in the direction perpendicular to the stretching force (see Biophys. J. 96, 1875 (2009)).
(DOC)

Figure S3 The predominant patterns for invading strand experiments. Reactions were done using surface bound 427/352 hybrid DNA with the complementary single stranded 427 nt DNA labeled with a polystyrene bead. (a). Type I: Initial BM increase, plateau, followed by a BM decrease to final product (28/46 = 61%). (b). Type II: Fluctuation around expected product Brownian motion amplitude (18/46 = 39%). Both types were successful events verified by the final Brownian motion amplitude localized within the yellow bar.
(DOC)

Figure S4 Control for outgoing strand experiments, using the 427/352 hybrid substrates, showed a very low percentage (<5%) of bead disappearance within 15 minutes. Out of these disappearing beads (<5%), none of them show an apparent BM change, most likely due to the stochastic detachment of either digoxigenin/anti-digoxigenin (surface/DNA) or biotin/streptavidin (bead/DNA) linkage. (a). Using homologous ssDNA with ATP, but no RecA. (b). Using homologous ssDNA with RecA, but no ATP. (c). Using heterologous ssDNA, with RecA and ATP. (d). Using only hybrid DNA anchored on surface without any other reagents.
(DOC)

Figure S5 Analysis for outgoing strand time-courses. Filled bars represent the case using ATP. Empty bars represent ATPγS. (a). A schematic time-course for the outgoing strand experiment includes a BM increase followed by bead disappearance. The duration between the plateau of bead BM till the time of bead disappearance is defined as τ_s_. b). The mean duration is 243±190 s and 211±150 s for ATPγS (N = 18) and ATP (N = 43), respectively.
(DOC)

Figure S6 The fraction of bead disappearance increases at longer reaction time in outgoing strand experiments. Filled squares represent the experiments with ATP (▪); filled circles represent ATPγS (•). Open squares represent control experiments without ATP (□); open circles represent controlled experiments without ssDNA and nucleotides (○); open triangles represent controlled experiments without RecA (△). Each point was the average of at least 3 experiments, with at least 100 tethers surveyed in each experiment.
(DOC)

Figure S7 The predominant patterns for outgoing strand experiments. Reactions were done using surface-bound, bead-labeled 427/352 hybrid DNA with complementary single-stranded incoming 427 nt DNA. (a). Initial BM increase, plateau, followed by a slow BM decrease before disappearance (51%). (b). No BM change. (20%) (c). Fluctuation around original hybrid DNA Brownian motion amplitude (18%). (d) A slow BM decrease before disappearance (11%). Similar to the BM patterns observed in the invading strand experiments (Figure S3), these patterns can be divided into type I (with apparent initial BM increase, plateau and slow BM decrease, as shown in **a** here), and type II (fluctuation around product BM value, as shown in **b**, **c**, and **d** here).
(DOC)

Figure S8 Time-courses of invading strand experiments. (a). Under limited RecA concentration (300 nM), insufficient to fully coat the all DNA, time-course shows the successful strand exchange product. (b). Under limited ATP concentration (500 µM) the time-course shows the successful strand exchange product. Both reactions were done using the 427/352 hybrid substrates.
(DOC)

Figure S9 The histograms of plateau BM value in the invading strand experiments. (a). Under sub-saturating ATP condition (500 µM), the combined (type I and type II) plateau value has the mean around 54.4 nm. (b). In the presence of ATPγS, RecA dissociation is inhibited, and the mean plateau value is higher (∼74.9 nm), suggesting longer filaments were involved.
(DOC)

Table S1 Primers used for the invading strand experiment.
(DOC)

Table S2 Primers used for the outgoing strand experiment.
(DOC)
