# Supplementary material for: Developing Single-Molecule TPM Experiments for Direct Observation of Successful RecA-Mediated Strand Exchange Reaction
Source: PLoS One. 2011 Jul 12;6(7):e21359. doi: 10.1371/journal.pone.0021359 (PMC3134461; doi:10.1371/journal.pone.0021359)
Supplement: Figure S1 — BM histograms of various DNA substrates, expressed by the standard derivation of bead centroid position. (a). 229 bp fully duplex DNA, 29.6±4.0 nm (N = 218). (b). 427 bp fully duplex DNA, 50.5±6.5 nm (N = 175). (c). 427/352 nt hybrid DNA, 36.6±4.7 nm (N = 78). (d). Fully RecA-coated 229 bp duplex DNA under ATPγS, 67.6±14.4 nm (N = 50). (e). Fully RecA-coated 427 bp dsDNA under ATPγS, 101.3±28.8 nm (N = 56). (DOC) [file pone.0021359.s001.doc]

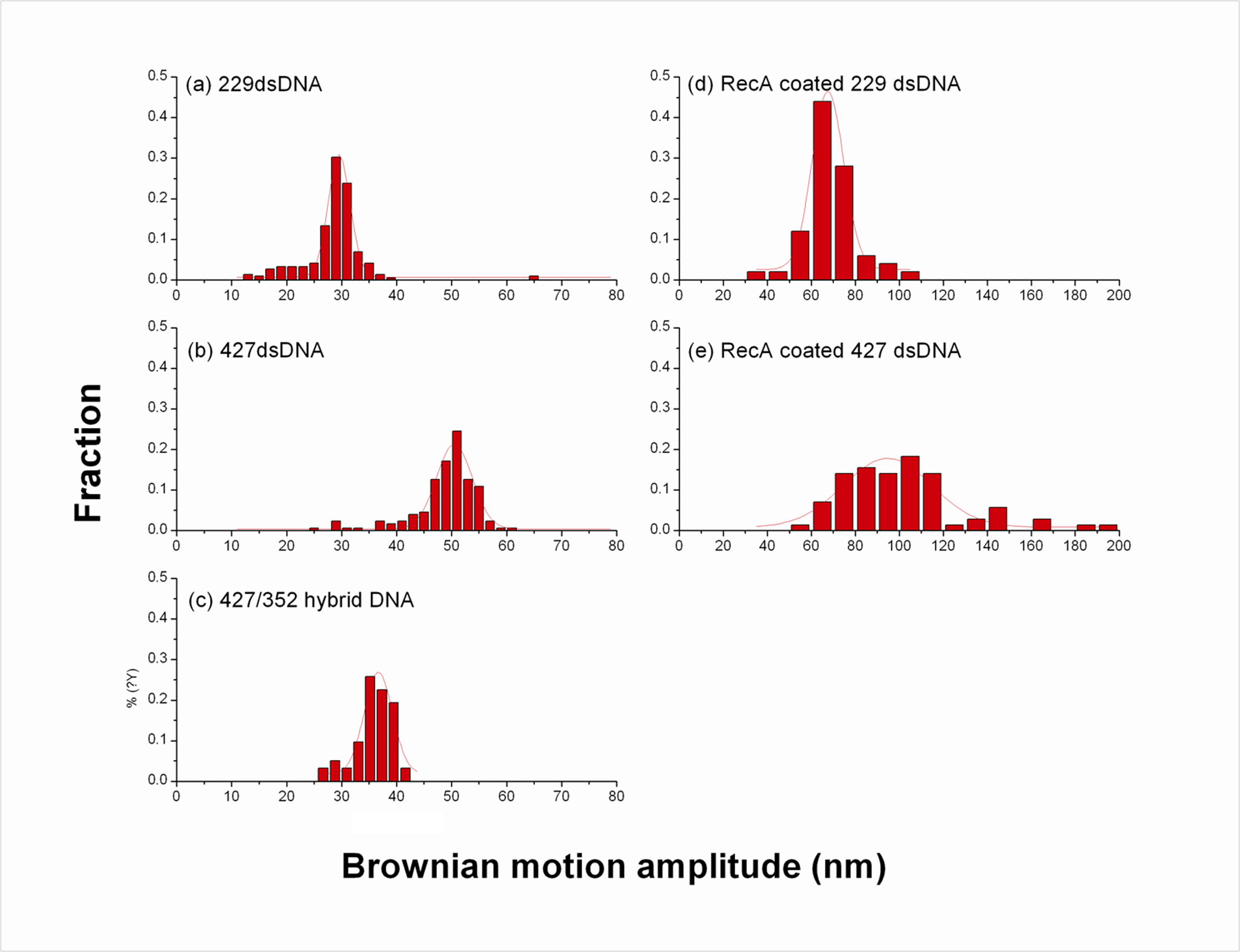


**Figure S1.** BM histograms of various DNA substrates, expressed by the standard derivation of bead centroid position. (a). 229 bp fully duplex DNA, 29.6 ± 4.0 nm (N=218). (b). 427 bp fully duplex DNA, 50.5 ± 6.5 nm (N=175). (c). 427/352 nt hybrid DNA, 36.6±4.7 nm (N=78). (d). Fully RecA-coated 229 bp duplex DNA under ATPγS, 67.6 ± 14.4 nm (N=50). (e). Fully RecA-coated 427 bp dsDNA under ATPγS, 101.3 ± 28.8 nm (N=56).
