# Supplementary material for: Developing Single-Molecule TPM Experiments for Direct Observation of Successful RecA-Mediated Strand Exchange Reaction
Source: PLoS One. 2011 Jul 12;6(7):e21359. doi: 10.1371/journal.pone.0021359 (PMC3134461; doi:10.1371/journal.pone.0021359)
Supplement: Figure S2 — The force-extension curve for a 836 bp dsDNA molecule tethered with 200 nm polystyrene bead done by applying a hydrodynamic force. Solid circles represent the force-extension curve for bare 836 bp dsDNA and empty circles represent that for RecA-coated 836 bp dsDNA using ATPγS. The solid curves are fitted to a worm-like chain model with fitted parameters for persistence length of 44.6±12.2 nm and 739.7±150.0 nm; for contour length of 280.3±2.4 and 475.3±2.8 nm for bare and RecA-coated dsDNA. The force was determined from the mean-squared displacement (MSD) of beads in the direction perpendicular to the stretching force (see Biophys. J. 96, 1875 (2009)). (DOC) [file pone.0021359.s002.doc]

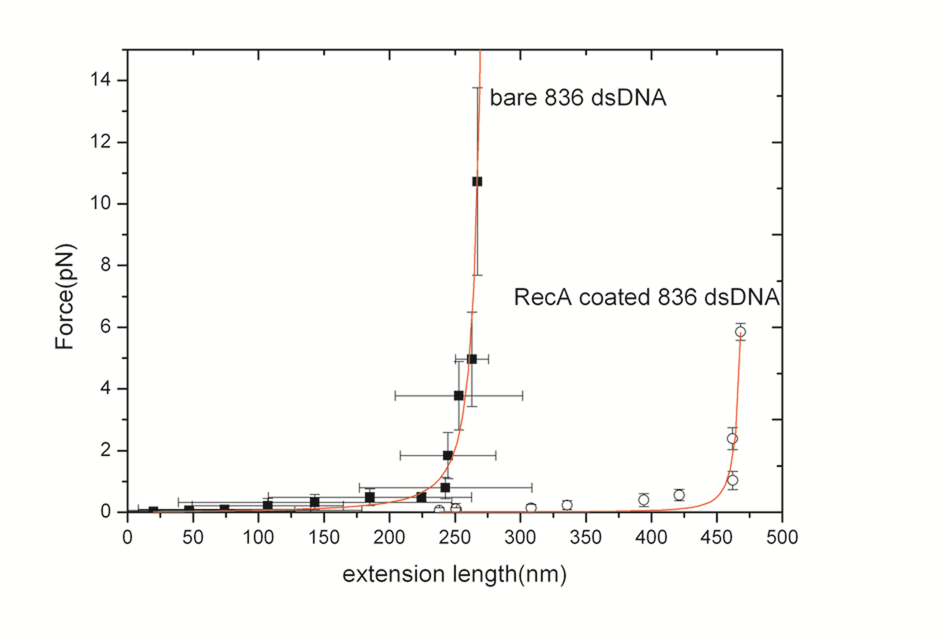


**Figure S2.** The force-extension curve for a 836 bp dsDNA molecule tethered with 200 nm polystyrene bead done by applying a hydrodynamic force. Solid circles represent the force-extension curve for bare 836 bp dsDNA and empty circles represent that for RecA-coated 836 bp dsDNA using ATPγS. The solid curves are fitted to a worm-like chain model with fitted parameters for persistence length of 44.6 ± 12.2 nm and 739.7 ± 150.0 nm; for contour length of 280.3 ± 2.4 and 475.3 ± 2.8 nm for bare and RecA-coated dsDNA. The force was determined from the mean-squared displacement (MSD) of beads in the direction perpendicular to the stretching force (see Biophys. J. 2009, 96, 1875).
