# Supplementary material for: Developing Single-Molecule TPM Experiments for Direct Observation of Successful RecA-Mediated Strand Exchange Reaction
Source: PLoS One. 2011 Jul 12;6(7):e21359. doi: 10.1371/journal.pone.0021359 (PMC3134461; doi:10.1371/journal.pone.0021359)
Supplement: Figure S3 — The predominant patterns for invading strand experiments. Reactions were done using surface bound 427/352 hybrid DNA with the complementary single stranded 427 nt DNA labeled with a polystyrene bead. (a). Type I: Initial BM increase, plateau, followed by a BM decrease to final product (28/46 = 61%). (b). Type II: Fluctuation around expected product Brownian motion amplitude (18/46 = 39%). Both types were successful events verified by the final Brownian motion amplitude localized within the yellow bar. (DOC) [file pone.0021359.s003.doc]

**Figure S3.** The predominant patterns for invading strand experiments. Reactions were done using surface bound 427/352 hybrid DNA with the complementary single stranded 427 nt DNA labeled with a polystyrene bead. (a). Type I: Initial BM increase, plateau, followed by a BM decrease to final product (28/46 = 61%). (b). Type II: Fluctuation around expected product Brownian motion amplitude (18/46 = 39%). Both types were successful events verified by the final Brownian motion amplitude localized within the yellow bar.
