# Supplementary material for: Developing Single-Molecule TPM Experiments for Direct Observation of Successful RecA-Mediated Strand Exchange Reaction
Source: PLoS One. 2011 Jul 12;6(7):e21359. doi: 10.1371/journal.pone.0021359 (PMC3134461; doi:10.1371/journal.pone.0021359)
Supplement: Figure S4 — Control for outgoing strand experiments, using the 427/352 hybrid substrates, showed a very low percentage (<5%) of bead disappearance within 15 minutes. Out of these disappearing beads (<5%), none of them show an apparent BM change, most likely due to the stochastic detachment of either digoxigenin/anti-digoxigenin (surface/DNA) or biotin/streptavidin (bead/DNA) linkage. (a). Using homologous ssDNA with ATP, but no RecA. (b). Using homologous ssDNA with RecA, but no ATP. (c). Using heterologous ssDNA, with RecA and ATP. (d). Using only hybrid DNA anchored on surface without any other reagents. (DOC) [file pone.0021359.s004.doc]

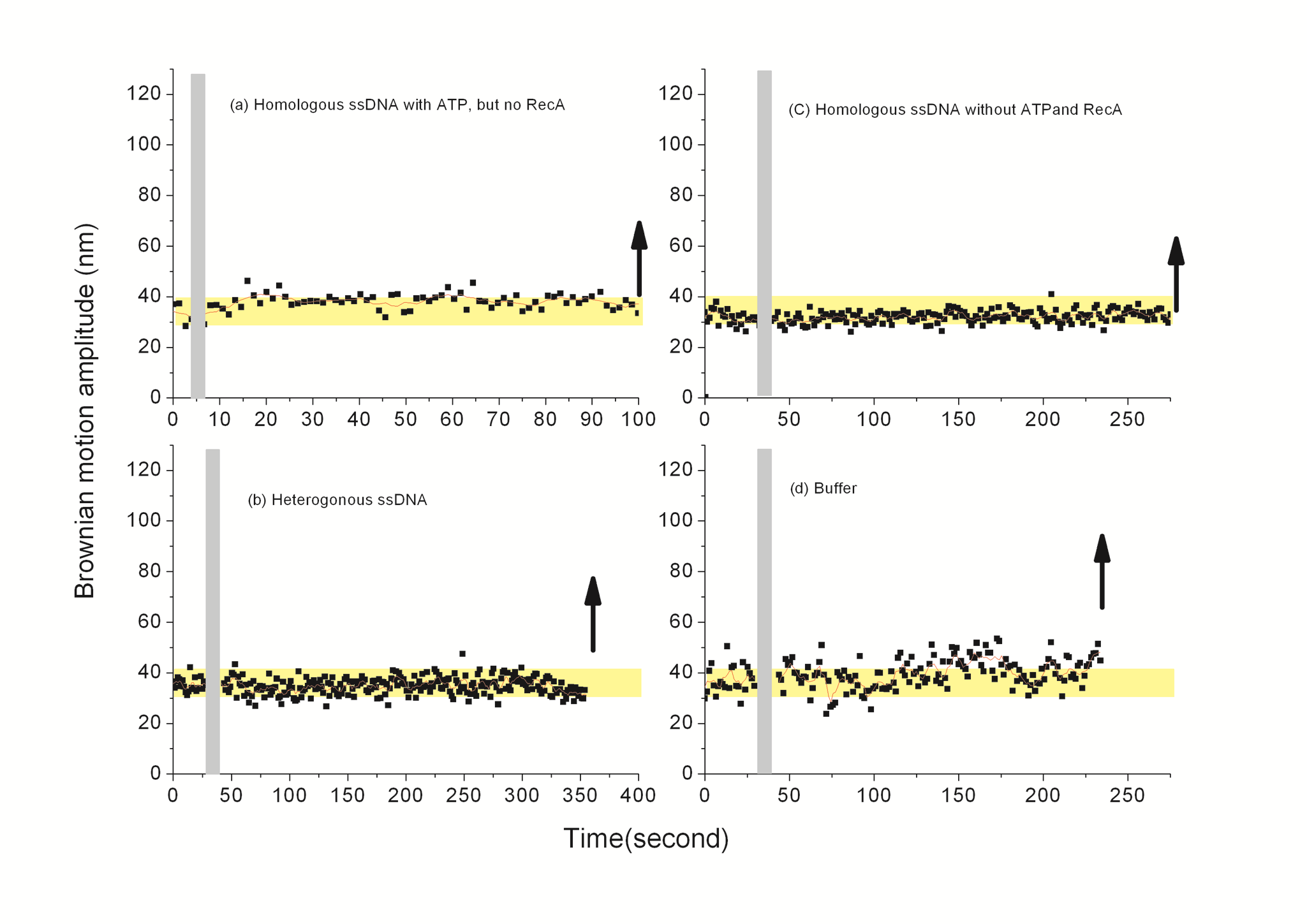


**Figure S4.** Control for outgoing strand experiments, using the 427/352 hybrid substrates, showed a very low percentage (< 5%) of bead disappearance within 15 minutes. Out of these disappearing beads (< 5%), none of them show an apparent BM change, most likely due to the stochastic detachment of either digoxigenin/anti-digoxigenin (surface/DNA) or biotin/streptavidin (bead/DNA) linkage. (a). Using homologous ssDNA with ATP, but no RecA. (b). Using homologous ssDNA with RecA, but no ATP. (c). Using heterologous ssDNA, with RecA and ATP. (d). Using only hybrid DNA anchored on surface without any other reagents.
