# Supplementary material for: Developing Single-Molecule TPM Experiments for Direct Observation of Successful RecA-Mediated Strand Exchange Reaction
Source: PLoS One. 2011 Jul 12;6(7):e21359. doi: 10.1371/journal.pone.0021359 (PMC3134461; doi:10.1371/journal.pone.0021359)
Supplement: Figure S5 — Analysis for outgoing strand time-courses. Filled bars represent the case using ATP. Empty bars represent ATPγS. (a). A schematic time-course for the outgoing strand experiment includes a BM increase followed by bead disappearance. The duration between the plateau of bead BM till the time of bead disappearance is defined as τs. b). The mean duration is 243±190 s and 211±150 s for ATPγS (N = 18) and ATP (N = 43), respectively. (DOC) [file pone.0021359.s005.doc]

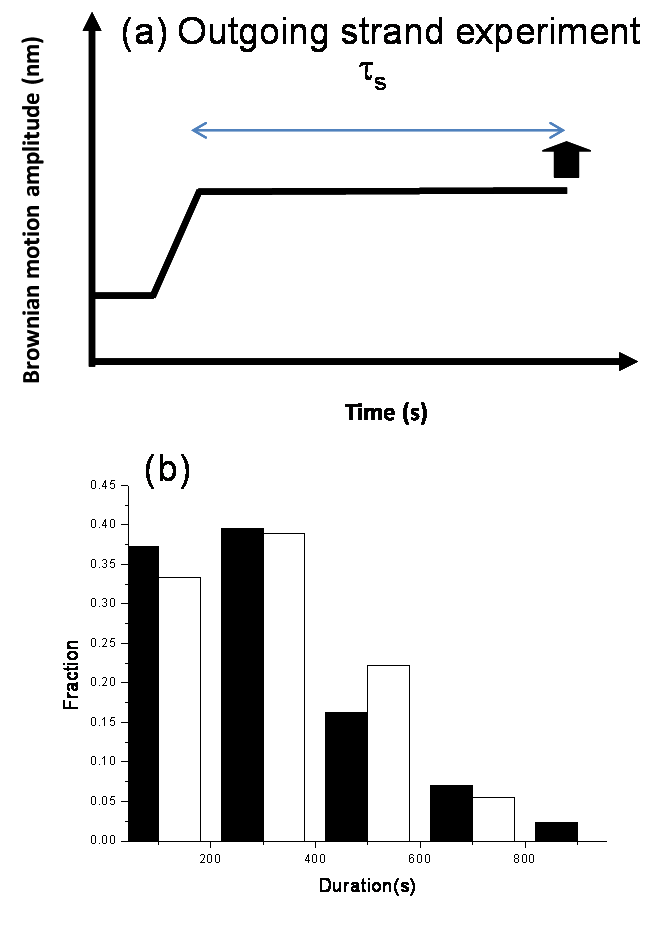


**Figure S5.** Analysis for outgoing strand time-courses. Filled bars represent the case using ATP. Empty bars represent ATPγS. (a). A schematic time-course for the outgoing strand experiment includes a BM increase followed by bead disappearance. The duration between the plateau of bead BM till the time of bead disappearance is defined as τ_s_. b). The mean duration is 243± 190 s and 211 ± 150 s for ATPγS (N=18) and ATP (N=43), respectively.
