# Supplementary material for: Developing Single-Molecule TPM Experiments for Direct Observation of Successful RecA-Mediated Strand Exchange Reaction
Source: PLoS One. 2011 Jul 12;6(7):e21359. doi: 10.1371/journal.pone.0021359 (PMC3134461; doi:10.1371/journal.pone.0021359)
Supplement: Figure S6 — The fraction of bead disappearance increases at longer reaction time in outgoing strand experiments. Filled squares represent the experiments with ATP (▪); filled circles represent ATPγS (•). Open squares represent control experiments without ATP (□); open circles represent controlled experiments without ssDNA and nucleotides (○); open triangles represent controlled experiments without RecA (△). Each point was the average of at least 3 experiments, with at least 100 tethers surveyed in each experiment. (DOC) [file pone.0021359.s006.doc]

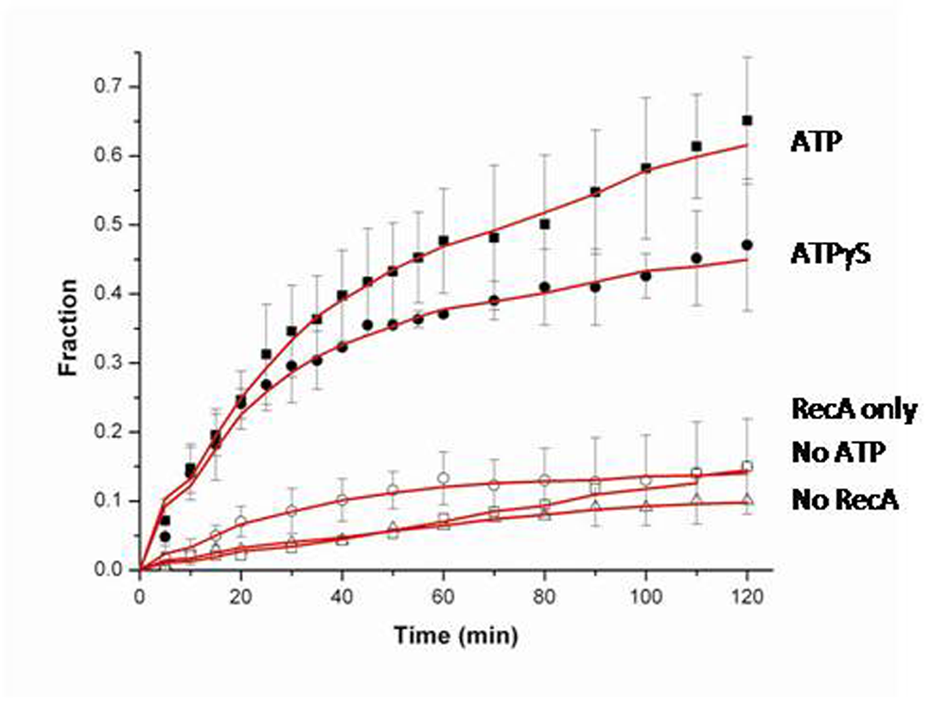


**Figure S6.** The fraction of bead disappearance increases at longer reaction time in outgoing strand experiments. Filled squares represent the experiments with ATP (■); filled circles represent ATPγS (●). Open squares represent control experiments without ATP (□); open circles represent controlled experiments without ssDNA and nucleotides (○); open triangles represent controlled experiments without RecA (△). Each point was the average of at least 3 experiments, with at least 100 tethers surveyed in each experiment.
