# Supplementary material for: Developing Single-Molecule TPM Experiments for Direct Observation of Successful RecA-Mediated Strand Exchange Reaction
Source: PLoS One. 2011 Jul 12;6(7):e21359. doi: 10.1371/journal.pone.0021359 (PMC3134461; doi:10.1371/journal.pone.0021359)
Supplement: Figure S7 — The predominant patterns for outgoing strand experiments. Reactions were done using surface-bound, bead-labeled 427/352 hybrid DNA with complementary single-stranded incoming 427 nt DNA. (a). Initial BM increase, plateau, followed by a slow BM decrease before disappearance (51%). (b). No BM change. (20%) (c). Fluctuation around original hybrid DNA Brownian motion amplitude (18%). (d) A slow BM decrease before disappearance (11%). Similar to the BM patterns observed in the invading strand experiments (Figure S3), these patterns can be divided into type I (with apparent initial BM increase, plateau and slow BM decrease, as shown in a here), and type II (fluctuation around product BM value, as shown in b, c, and d here). (DOC) [file pone.0021359.s007.doc]

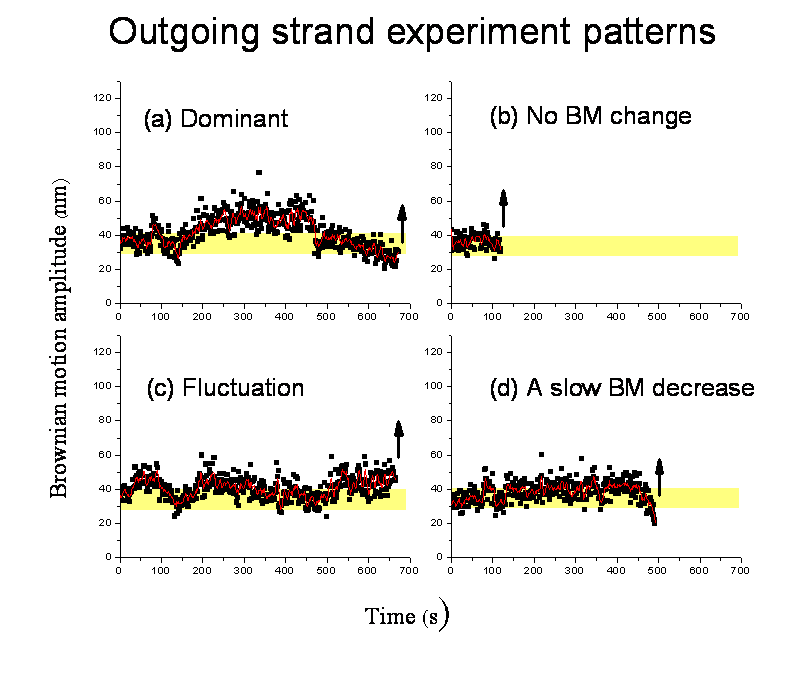


**Figure S7.** The predominant patterns for outgoing strand experiments. Reactions were done using surface-bound, bead-labeled 427/352 hybrid DNA with complementary single-stranded incoming 427 nt DNA. (a). Initial BM increase, plateau, followed by a slow BM decrease before disappearance (51%). (b). No BM change. (20%) (c). Fluctuation around original hybrid DNA Brownian motion amplitude (18%). (d) A slow BM decrease before disappearance (11%). Similar to the BM patterns observed in the invading strand experiments (Figure S3), these patterns can be divided into type I (with apparent initial BM increase, plateau and slow BM decrease, as shown in **a** here), and type II (fluctuation around product BM value, as shown in **b**, **c**, and **d** here).
