# Supplementary material for: Developing Single-Molecule TPM Experiments for Direct Observation of Successful RecA-Mediated Strand Exchange Reaction
Source: PLoS One. 2011 Jul 12;6(7):e21359. doi: 10.1371/journal.pone.0021359 (PMC3134461; doi:10.1371/journal.pone.0021359)
Supplement: Figure S8 — Time-courses of invading strand experiments. (a). Under limited RecA concentration (300 nM), insufficient to fully coat the all DNA, time-course shows the successful strand exchange product. (b). Under limited ATP concentration (500 µM) the time-course shows the successful strand exchange product. Both reactions were done using the 427/352 hybrid substrates. (DOC) [file pone.0021359.s008.doc]

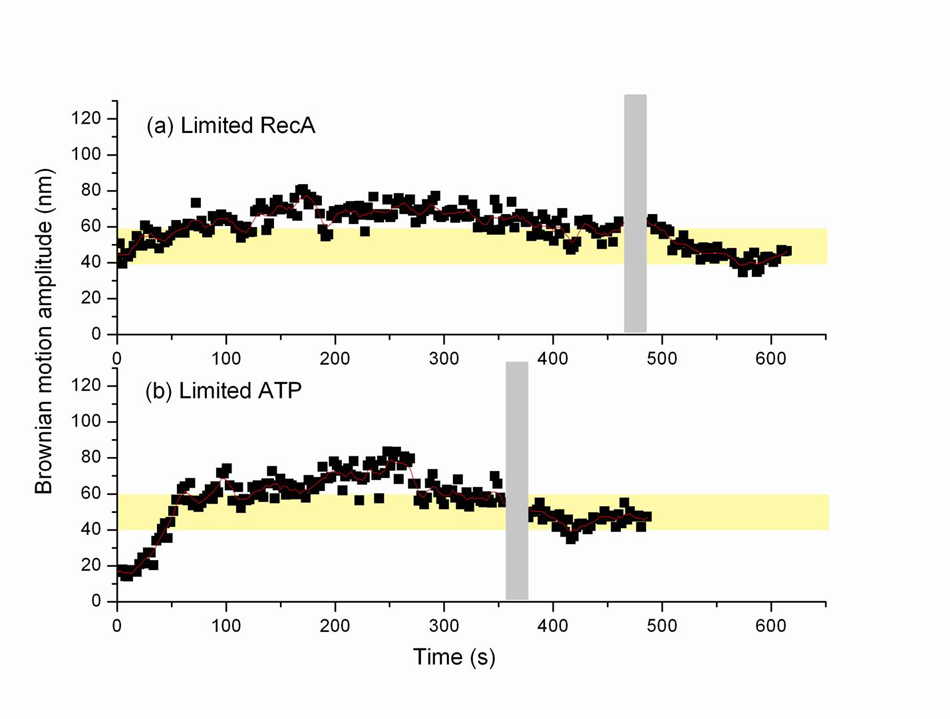


**Figure S8.** Time-courses of invading strand experiments. (a). Under limited RecA concentration (300 nM), insufficient to fully coat the all DNA, time-course shows the successful strand exchange product. (b). Under limited ATP concentration (500 μM) the time-course shows the successful strand exchange product. Both reactions were done using the 427/352 hybrid substrates.
