# Supplementary material for: Developing Single-Molecule TPM Experiments for Direct Observation of Successful RecA-Mediated Strand Exchange Reaction
Source: PLoS One. 2011 Jul 12;6(7):e21359. doi: 10.1371/journal.pone.0021359 (PMC3134461; doi:10.1371/journal.pone.0021359)
Supplement: Figure S9 — The histograms of plateau BM value in the invading strand experiments. (a). Under sub-saturating ATP condition (500 µM), the combined (type I and type II) plateau value has the mean around 54.4 nm. (b). In the presence of ATPγS, RecA dissociation is inhibited, and the mean plateau value is higher (∼74.9 nm), suggesting longer filaments were involved. (DOC) [file pone.0021359.s009.doc]

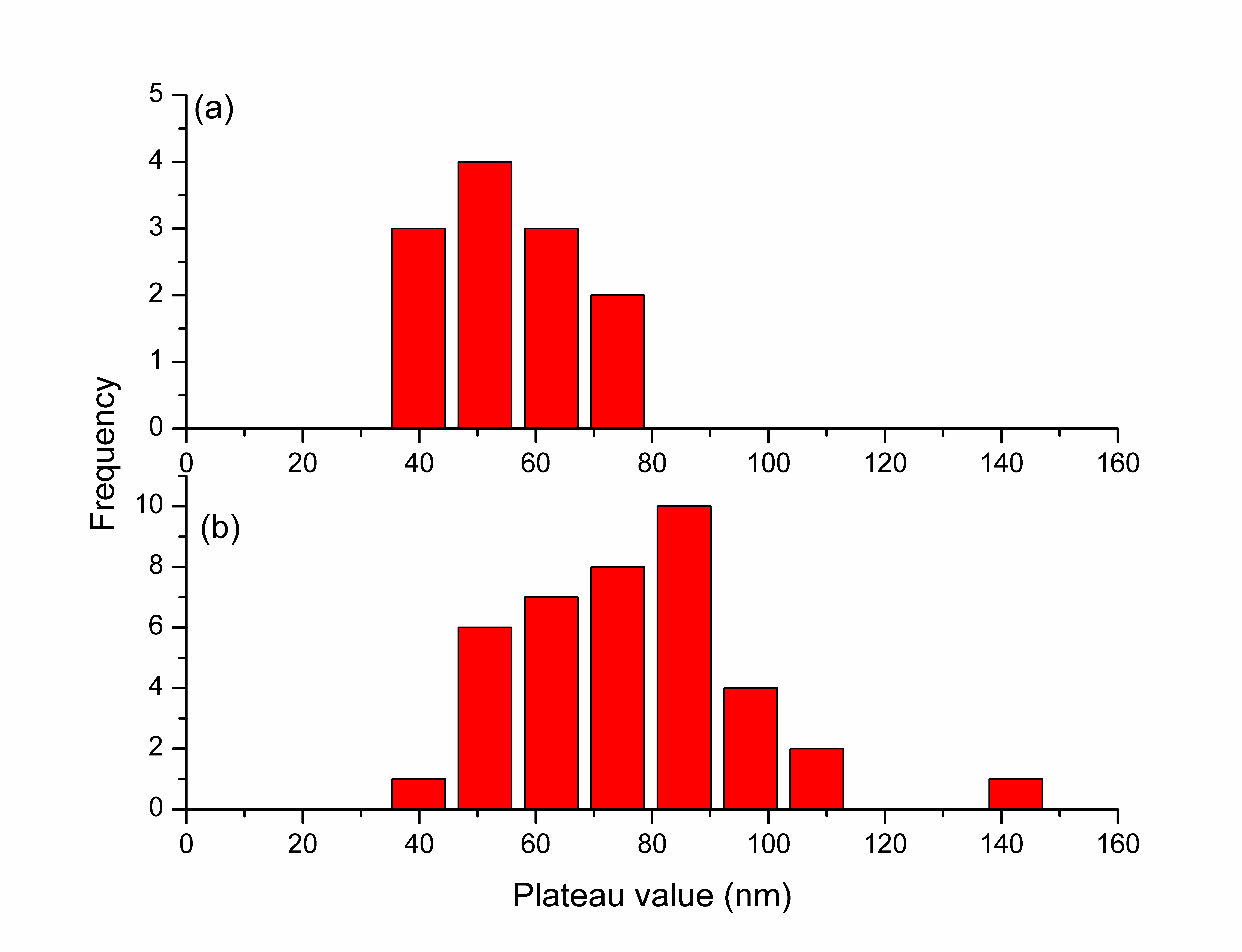


**Figure S9.** The histograms of plateau BM value in the invading strand experiments. (a). Under sub-saturating ATP condition (500 μM), the combined (type I and type II) plateau value has the mean around 54.4 nm. (b). In the presence of ATPγS, RecA dissociation is inhibited, and the mean plateau value is higher (~ 74.9 nm), suggesting longer filaments were involved.
