# Supplementary material for: Developing Single-Molecule TPM Experiments for Direct Observation of Successful RecA-Mediated Strand Exchange Reaction
Source: PLoS One. 2011 Jul 12;6(7):e21359. doi: 10.1371/journal.pone.0021359 (PMC3134461; doi:10.1371/journal.pone.0021359)
Supplement: Table S2 — Primers used for the outgoing strand experiment. (DOC) [file pone.0021359.s011.doc]

Table S2. Primers used for outgoing strand experiment

| DNA substrate | template | Primer sequence |
| --- | --- | --- |
| 427/352 hybrid dsDNA | 3χF3χH | 5′-DigN-ACTACGATACGGGAGGGC  5′-Phos-CGGATGGCATGACAGTAAG  5′-Bio-TGAGTGATAACACTGCGGC  5-Phos-CCCAGTGCTGCAATGATACC |
| 427 ssDNA | 5′-Phos-ACTACGATACGGGAGGGC  5′-OH-CGGATGGCATGACAGTAAG |
| Non-homologous  427 ssDNA | 5′-Phos-GATGGCGCCCAACAGTCCC  5′-OH-GGCATAGGCTTGGTTATGC |
